# BRCA1-BARD1 regulates transcription through modulating topoisomerase IIβ

**DOI:** 10.1098/rsob.210221

**Published:** 2021-10-06

**Authors:** Heeyoun Bunch, Jaehyeon Jeong, Keunsoo Kang, Doo Sin Jo, Anh T. Q. Cong, Deukyeong Kim, Donguk Kim, Dong-Hyung Cho, You Mie Lee, Benjamin P. C. Chen, Matthew J. Schellenberg, Stuart K. Calderwood

**Affiliations:** ^1^ Department of Applied Biosciences, College of Agriculture and Life Sciences, College of Pharmacy, Kyungpook National University, Daegu 41566, Republic of Korea; ^2^ School of Applied Biosciences, College of Agriculture and Life Sciences, College of Pharmacy, Kyungpook National University, Daegu 41566, Republic of Korea; ^3^ School of Life Sciences, BK21 Four KNU Creative Bioresearch Group, College of Pharmacy, Kyungpook National University, Daegu 41566, Republic of Korea; ^4^ Vessel-Organ Interaction Research Center, VOICE (MRC), Department of Molecular Pathophysiology, College of Pharmacy, Kyungpook National University, Daegu 41566, Republic of Korea; ^5^ Department of Microbiology, College of Natural Sciences, Dankook University, Cheonan 31116, Republic of Korea; ^6^ Department of Biochemistry and Molecular Biology, Mayo Clinic, Rochester, MN 55905, USA; ^7^ Department of Radiation Oncology, University of Texas Southwestern Medical Center, Dallas, TX 75390, USA; ^8^ Department of Radiation Oncology, Beth Israel Deaconess Medical Center, Harvard Medical School, Boston, MA 02115, USA

**Keywords:** BRCA1-BARD1 complex, topoisomerase IIβ, transcription-coupled DNA break, gene regulation, stimulus-inducible transcriptional activation

## Abstract

RNA polymerase II (Pol II)-dependent transcription in stimulus-inducible genes requires topoisomerase IIβ (TOP2B)-mediated DNA strand break and the activation of DNA damage response signalling in humans. Here, we report a novel function of the breast cancer 1 (BRCA1)-BRCA1-associated ring domain 1 (BARD1) complex in this process. We found that BRCA1 is phosphorylated at S1524 by the kinases ataxia-telangiectasia mutated and ATR during gene activation, and that this event is important for productive transcription. Our biochemical and genomic analyses showed that the BRCA1-BARD1 complex interacts with TOP2B in the *EGR1* transcription start site and in a large number of protein-coding genes. Intriguingly, the BRCA1-BARD1 complex ubiquitinates TOP2B, which stabilizes TOP2B binding to DNA while BRCA1 phosphorylation at S1524 controls the TOP2B ubiquitination by the complex. Together, these findings suggest the novel function of the BRCA1-BARD1 complex in the regulation of TOP2B and Pol II-mediated gene expression.

## Graphical abstract

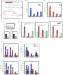

## 1. Introduction

Transcription is the first and most immediate step in gene expression, but also contributes to genomic instability. The innate genotoxic mechanisms appearing during transcription include R-loop-mediated susceptibility of the non-template DNA and the collision of replication–transcription machineries on chromosomes, which result in single-strand or double-strand breakage (DSB) of DNA [1,2]. In addition, a few studies in the past decade have reported the formation of DSB, a so-called programmed DNA break, during transcription, in which DSB is induced by stimuli such as steroid-hormone receptor binding and neurotransmitter- and serum-induced gene activation [3–8]. In the latter cases, topoisomerase IIβ (TOP2B) is responsible for generating the DSB [3,6,7]. It has been suggested that TOP2B-mediated DSBs are persistent rather than momentary and instantaneously reversed as it would be expected for typical topoisomerase-mediated topological resolution [9]. This speculation is based on the fact that the catalytic activity of TOP2B during transcriptional activation induces DNA damage response (DDR) signalling [9,10]. Importantly, DSB is required for efficient transcription because inhibiting either TOP2B or major DDR kinases, including DNA-dependent protein kinase (DNA-PK) and ataxia-telangiectasia mutated (ATM), has been shown to deregulate polymerase II (Pol II) translocation from early elongation to processive elongation; this in turn interferes with productive RNA synthesis in *HSP70*, enhancer RNA, and the serum- and oestrogen receptor-activated genes in humans [3,4,6–8]. These observations suggest a potential innate cause for genomic ageing or mutability attributed to gene expression during transcription that is an inevitable process in mediating growth, differentiation and maintenance of the cells and organs of any organism. In addition, the data signify that the transcription-coupled DNA repair system plays a crucial and essential role in protecting the genome from accumulating instability.

Repair of most genomic DSBs involves two major pathways: the non-homologous end joining (NHEJ) and homology-directed repair (HDR) [11]. 53BP1 and breast cancer 1 (BRCA1) perform completing roles in the selection of NHEJ versus HDR pathways for the repair of a DNA break [12,13]. Because of the requirement for an intact homologous strand, HDR is thought to be more restricted to the S/G_2_ cell cycle phase in higher eukaryotes, while NHEJ predominates in the G_1_ phase of the cell cycle [14,15]. However, BRCA1 appears to be involved in NHEJ as well and reportedly participates in precise NHEJ during the G_1_ phase [16]. In addition, recent studies have found that BRCA1 is recruited to R-loop and Pol II pausing sites [17–19], and plays critical roles in removing and repairing TOP2-DNA adducts in replication and oestrogen-induced transcription [20–22]. The expression of BRCA1 is maintained at a relatively low level in the cell, and BRCA1 activity is regulated by post-translational modifications, including phosphorylation, methylation, sumoylation and ubiquitination (https://www.uniprot.org/uniprot/P38398). Upon DNA damage, BRCA1 is phosphorylated at multiple residues, including serines 1387, 1423, 1457 and 1524 by ATM [23,24]. BRCA1 often functions in complex with BRCA1-associated ring domain 1 (BARD1) as an E3 ubiquitin ligase during DNA repair and cell proliferation [25–29]. BRCA1-BARD1 heterodimers are ubiquitinated, which stimulates the ubiquitin ligase activity of this protein complex [30]. Thus far, H2A, NF2 and oestrogen receptor *α* have been identified as ubiquitination substrates of the BRCA1-BARD1 complex [28,29,31].

Transcription shows markedly increased activity during the G_1_ phase to mediate the expression of the proteins and biomolecules required for genome replication in the S phase and later steps in cell proliferation. This process is initiated by human immediate early genes (hIEGs), which are transcribed as cells transit from the G_0_ to the G_1_ phase. Many such genes are transcription factors and proto-oncogenes, such as *JUN*, *FOS*, *MYC* and *EGR1*. These genes are expressed rapidly upon the receipt of cell proliferation signals, and their transcription requires the mechanism underlying Pol II promoter-proximal pausing (Pol II pausing), followed by pause release [3,32]. In the resting state of transcription, Pol II pausing occurs at approximately positions +25 to +100 from the transcription start site (TSS) of a large number of protein-coding and non-coding genes in metazoan cells [4,33–37]. Gene activation involves the release of Pol II from the pausing site and the resumed production of a full-length transcript [4,37–40]. It has been reported that TOP2B-mediated DSB and DDR signalling are accompanied by and required for Pol II pause release and gene activation in hIEGs [3,7,41]. This finding raises important questions regarding how TOP2B is regulated and TOP2B-mediated DSB is repaired during transcription.

In this study, we have investigated whether BRCA1 regulates the transcription of stimulus-inducible genes and, in particular, the functional relationship of this protein with TOP2B during transcriptional pausing and activation. Through biochemical and cell-based analyses, we found that the BRCA1-BARD1 complex is important for the expression of the *EGR1* gene, a representative hIEG that uses Pol II pausing for gene regulation. Upon serum induction, ATM and ATR phosphorylate BRCA1 at S1524, which is a well-characterized residue to be phosphorylated during DDR signalling [23,24,42]. The phosphorylation of BRCA1 activates transcription in hIEGs. The BRCA1-BARD1 complex functions in TOP2B stabilization as indicated by findings that BARD1 KD reduces TOP2B protein levels. Genomic analyses showed the colocalization of BRCA1 and TOP2B in a large number of protein-coding genes in humans, suggesting their widespread functional involvement. In addition, we found that TOP2B binds to a fragment including –132 to +62 of *EGR1* TSS with high affinity (*K*_d_ = 59.9 ± 6.1 nM) and that BARD1 colocalizes with TOP2B within a 118 bp portion of this DNA segment (–132 to –15). Importantly, we identified the E2 enzymes UBCH13/MMS2 and UBCH5b that are engaged with BRCA1-BARD1 to ubiquitinate TOP2B and we showed that the ubiquitination event enhances TOP2B binding to the *EGR1* TSS. Our biochemical analyses suggested that the phosphorylation status of BRCA1 at S1524 controls the BRCA1-BARD1 complex–TOP2B functional interaction: the non-phosphorylatable BRCA1 S1524A mutant ubiquitinates TOP2B more effectively than WT or a phosphomimetic BRCA1, which leads to a stronger association between TOP2B and *EGR1* TSS. Together, these results suggest a novel role for BRCA1-BARD1 complex as a transcription factor and in the regulation of TOP2B in hIEG transcription.

## 2. Material and methods:

### 2.1. Cell culture and experimental conditions

HEK293 cells were grown in a complete medium, composed of DMEM (Corning), supplemented with 10% FBS (Gibco) and 1% penicillin/streptomycin solution (P/S, Gibco). For serum induction experiments, HEK293 cells were grown to about 80% confluence. The cells were incubated in DMEM, including 0.1% FBS and 1% P/S solution, for 17.5 h and then were induced using serum through incubation in DMEM, supplemented with 18% FBS and 1% P/S solution. After serum induction, cells were collected at the corresponding time points listed in the figures. For the ATM and ATR inhibitor experiment, HEK293 cells were incubated in the 0.1% serum media for 17.5 h. The media were exchanged with the 0.1% serum media including KU55933 (Abcam, ab120637), VE821 (Sigma, SML-1415) or caffeine (Sigma, C0750) at a final concentration of 10 µM, 1 µM or 3 mM in 0.1% DMSO (for KU55933 and VE821) and 4% water (for caffeine) of the total media volume. The cells were incubated for 1 h before serum induction for 15 min with 18% serum media, including the chemicals at the same final concentration. Control cells were prepared side-by-side using DMSO or water only at the same final concentration.

### 2.2. Cell transfection

HEK293 cells were grown to approximately 70% confluence in complete media. The media were exchanged with the complete media without antibiotics immediately before transfecting the cells with scrambled (no. 6568, Cell Signaling) or BARD1 siRNA duplexes (SR300400, Origene) and BRCA1-targeting siRNA species (sc-29219, Santa Cruz Biotechnology) dissolved in serum-free DMEM using Lipofectamine 2000 (Invitrogen) according to the manufacturer's instructions. The cells were collected after 48 h or 72 h incubation for RNA or protein analyses, respectively, or were subjected to serum starvation/induction for chromatin immunoprecipitation assays, western blotting or immunoprecipitation.

### 2.3. RNA quantification

RNA molecules were extracted using RNeasy kit (Qiagen) following the manufacturer's instructions. From each experimental condition, 0.6 or 1 µg extracted RNA was converted into cDNA by reverse transcription using a High Capacity cDNA Reverse Transcription kit (Promega A3500) or a ReverTra Ace qPCR RT Master Mix (Toyobo). RT-qPCR was conducted with equal amounts of resultant cDNAs and indicated primers (electronic supplementary material, table S1) using platinum tag DNA polymerase high fidelity (Invitrogen) under thermal cycling for 2 min at 94°C followed by 25 cycles of 20 s at 94°C, 30 s at 55°C and 30 s at 68°C or through GO taq polymerase (Promega) for 2 min at 95°C followed by 25 cycles of 30 s at 95°C, 30 s at 55°C and 30 s at 72°C. Real-time quantitative PCR (qRT-PCR) was performed with a CFX96 real-time PCR detection system (Bio-Rad), thermal cycler dice real-time system III (Takara) or StepOnePlus real-time PCR system (Applied Biosystems) using β-actin as an internal control under thermal cycling as 1 min at 95°C followed by 45 cycles of 15 s at 95°C, 15 s at 55°C and 1 min at 72°C. The primers used for the study are listed in the electronic supplementary material, table S1. The results are presented as means of SEMs after normalization.

### 2.4. DNA templates

The DNA template of *EGR1* promoter and early transcript, including –423 to +332, was amplified from HeLa nuclear extract (NE) using a pair of primers (electronic supplementary material, table S1). The amplified product was cloned into a pCR-Blunt-TOPO plasmid, called pTOPO-EGR1. The biotinylated template was generated via PCR using the cloned vector as a template and a set of primers, one conjugated with biotin at the 5′ end (electronic supplementary material, table S1). The PCR product was sequence-verified, gel-extracted and purified using the Qiaquick gel extraction kit (Qiagen) before further experimentation. The mutant pTOPO-EGR1 vectors including the altered sequences between +141 and +160 and a point substitution at +69 that generated a SfoI site were synthesized via Quikchange site-directed mutagenesis, using pairs of primers (electronic supplementary material, table S1) incorporating these mutations. All primers that were designed and used in the study were purchased from Integrated DNA Technology, and their sequences are listed in the electronic supplementary material, table S1.

### 2.5. Proteins, expression vectors and purification

A full-length BRCA1 was amplified from a BRCA1 expression vector, pcBRCA1-385, provided by Dr Mike Erdos at the National Institutes of Health and cloned into bacterial expression vector pET17b including a His^6^ tag with a pair of primers (electronic supplementary material, table S1) to produce pET-WT-BRCA1. The pET17b vectors expressing mutant BRCA1 species S1524A and S1524D (pET-SA-BRCA1 and pET-SD-BRCA1) were generated by Quikchange site-directed mutagenesis, using pairs of primers (electronic supplementary material, table S1) that incorporated the desired amino acid substitutions. A full-length coding sequence of BARD1 was amplified from BARD1 cDNA (HG15850-CH, Sino Biological) and cloned into another bacterial expression vector, pET29b including His^6^ tag in the C-terminal. A full-length coding sequence of TOP2B was amplified from our cDNA library of HEK293 cells. Our bacterial-derived TOP2B construct encoded the N-terminal 566 amino acids. It was cloned to pET21a, including His^6^ in the N-terminal domain. The primers used to amplify and clone the coding sequences of BARD1 and TOP2B are listed in the electronic supplementary material, table S1. These five protein expression vectors were sequence-verified, and each was transformed into BL21. The His^6^-tagged proteins were purified using Ni beads (Invitrogen). Protein expression was induced at 20°C for 16 h using 0.5–1 mM IPTG as a final concentration (electronic supplementary material, figure S1). The *E. coli* cells expressing WT-, SA- and SD-BRCA1 were harvested and lysed with the xTractor bacterial cell lysis buffer (Clontech) and sonication. The Ni wash buffers included 10 mM and 25 mM imidazole, 0.5 M NaCl, 10 mM Tris-HCl pH 7.6, 0.5% Triton X-100 and 10% glycerol. The wash was performed—twice with 10 mM imidazole wash buffer, and then twice with 25 mM buffer for BRCA1 and four times with 25 mM buffer for BARD1 and TOP2B. Bead-bound proteins were eluted with 500 mM imidazole buffer including 0.02% NP40. The eluted proteins were subjected to SDS-PAGE and immunoblotting to be verified. All protein purification buffers included freshly added protease inhibitors, 1 mM benzamidine (Sigma), 0.25 mM PMSF (Sigma), aprotinin (Sigma A6279, 1 : 1000) and 1 mM Na-metabisulfite (Sigma). DNA encoding full-length human TOP2B (amino acids 1-1621) was amplified by PCR from pcDNA6.2/C-YFPDest-TOP2B [43] with primers that incorporate a HRV3C protease site at the N-terminus of TOP2B and cloned into the vector pmCentr2 using ligation-independent cloning. LR Clonase II (Thermofisher) was used to transfer the HRV3C-TOP2B insert into pcDNA6.2/N-YFPDest to generate the YFP-TOP2B plasmid, transformed into the Stbl3 *E. coli* strain (Thermofisher) and purified using a Plasmid DNA Gigaprep kit (Zymo). The insert was fully sequenced to confirm the absence of mutations (see electronic supplementary material, table S1 for primer sequences). YFP-TOP2B was transfected into HEK293F cells in Hyclone TransFx media (Cytiva) using PEI MAX (linear polyethylene imine, MW 40 000; Polysciences) and purified using the YFP-tag system [44]. HEK293F cell pellets were lysed in 36 ml lysis buffer (50 mM Tris-HCl pH 8.0, 400 mM NaCl, 0.1% (v/v) NP-40 substitute (Sigma) and 1 mM TCEP [Tris(2-carboxyethyl)phosphine, Sigma]), supplemented with complete EDTA-free protease inhibitor cocktail (Roche) and sonicated with three cycles of 5 s using a Branson sonicator set to 55% power, followed by 30 s cooling periods. Crude lysate was centrifuged at 25 000 x g for 10 min, then passed over a column with pre-equilibrated anti-GFP/YFP sepharose resin. The resin was washed six times with lysis buffer (supplemented with 100 nM USP2 (purified in-house from Addgene plasmid 36894) for de-ubiquitinated TOP2B), then three times in ATP wash buffer (50 mM Tris-HCl pH 8.0, 200 mM NaCl, 2 mM MgCl_2_, 0.05% Tween-20, 1 mM TCEP and 2 mM ATP; with 100 nM USP2 for de-ubiquitinated TOP2B) followed by a 15 min incubation at room temperature to remove HSP70 contaminant. Resin was then washed three times with cold size-exclusion buffer (20 mM Tris-HCl pH 7.5, 500 mM NaCl and 1 mM TCEP), then 1.5 column volumes of size-exclusion buffer supplemented with 45 µg ml^−1^ HRV3C protease. Column was capped tightly and incubated overnight to cleave TOP2B from the YFP-tag. TOP2B was eluted the next day by washing the resin with size-exclusion and diluted in three volumes of low salt buffer (20 mM Tris-HCl pH 7.5, 1 mM DTT), loaded onto a 6 ml SOURCE 15S column (GE Healthcare) and eluted with a linear gradient of 0–50% high-salt buffer (20 mM Tris-HCl pH 7.5, 1 M NaCl). Fractions containing TOP2B were pooled and concentrated to 2 ml volume by ultrafiltration (Amicon) and run on a Superdex200 16/60 column (GE) in size-exclusion buffer. Fractions containing TOP2B were pooled, concentrated by ultrafiltration and buffer exchanged into TOP2 storage buffer (20 mM HEPES pH 7.5, 500 mM NaCl, 1 mM TCEP and 25% (v/v) glycerol. TOP2 decatenase activity was assayed in 10 µl reactions that contained 200 ng kDNA (Topogen) and 1.7–50 nM TOP2B according to the manufacturer's protocol.

### 2.6. HeLa nuclear extract preparation

HeLa nuclei were provided by Taatjes laboratory (University of Colorado, Boulder). The nuclei were dissolved with 0.9 volumes of Buffer C (20 mM HEPES pH 7.9, 25% glycerol, 420 mM NaCl, 1.5 mM MgCl_2_ and 0.2 mM EDTA) while stirring at 4°C. The mixture was dounced 20 times with pestle B before being stirred gently for 30 min at 4°C. Then, the homogenized solution was centrifuged for 30 min at 13 000 rpm at 4°C. The supernatant was dialysed against Buffer D (20 mM HEPES pH 7.9, 20% glycerol, 100 mM KCl, 2 mM MgCl_2_ and 0.2 mM EDTA) to conductivity 100–150 mM [4]. Buffers C and D included the fresh protease inhibitors described above and 1 mM DTT (complete protease inhibitors). The resulting HeLa NE was validated for protein quality and concentration by western blotting for probing nuclear proteins, including RNA polymerase II and BRCA1 and Bradford assays in comparison with a reference HeLa NE, provided by Taatjes laboratory.

### 2.7. Immobilized template assay and transcription assay

The experimental procedure for immobilized template and transcription assays is identical to that of our previous report, except for the method of quantifying nascent RNAs synthesized *in vitro* [4]. Dynabeads M-280 Streptavidin (Invitrogen) was prepared with 2X B&W buffer (10 mM Tris-HCl, pH 7.5, 1 mM EDTA and 2 M NaCl) and incubated with biotin-conjugated *EGR1* template DNA (–423 to +332) at 10 ng DNA/μL beads. The template-conjugated beads were washed with 1X B&W buffer and 0.1 M Buffer D1 (20 mM HEPES, 20% glycerol, pH 7.6, pH 7.9, 0.1 mM EDTA and 100 mM KCl). Then 120 ng immobilized template was mixed with TF buffer (12.5 ng µl^−1^ dI-dC, 0.075% NP40, 5 mM MgCl_2_, 250 ng µl^−1^ BSA, 12.5% glycerol, 100 mM KCl, 12.5 mM HEPES, pH 7.6, 62.5 µM EDTA and 10 µM ZnCl_2_) for pre-incubation with purified WT or mutant BRCA1 protein. The resultant template-protein complex was pulled-down using a magnet stand (Invitrogen) and resuspended in NE buffer (17.5 ng µl^−1^ dI-dC, 0.1% NP40, 7.5 mM MgCl_2_, 1.25 µg µl^−1^ BSA, 8.7% glycerol, 8.7 mM HEPES, pH 7.6, 44 µM EDTA, 130 mM KCl and 10 µM ZnCl_2_). HeLa NE and purified recombinant WT or mutant BRCA1 protein when indicated as T1 was added at 100 µg/reaction and incubated with agitation for 30 m at room temperature (RT) to assemble PIC. The template-protein complex was washed briefly with a 10-bead volume of TW buffer (13 mM HEPES, pH 7.6, 13% glycerol, 60 mM KCl, 7 mM MgCl_2_, 7 mM DTT, 100 µM EDTA, 0.0125% NP40 and 10 µM ZnCl_2_) and then resuspended in transcription buffer I (13 mM HEPES, pH 7.6, 13% Glycerol, 60 mM KCl, 7 mM MgCl_2_, 10 µM ZnCl_2_, 7 mM DTT, 100 µM EDTA, 15 ng µl^−1^ dI-dC and 10 mM creatine phosphate). For transcription assay, a mixture of NTP in final concentrations of 250 µM A/G/C/U was added to initiate Pol II to polymerize mRNA molecules at 30°C. When T2 was indicated, purified WT or mutant BRCA1 protein was introduced, both during the PIC assembly and after 3 min of NTP addition. The polymerization reaction was allowed for 30 min. Then, 1.5 Kunitz unit DNase I (Qiagen) was added to the reaction and allowed to sit for an additional 15 min to remove the template DNA. All reactions were terminated with 5 volumes 1.2 X Stop buffer (0.6 M Tris-HCl, pH 8.0, 12 mM EDTA and 100 µg ml^−1^ tRNA). The pellet fraction, including the magnetic bead-template DNA complex, was removed. The supernatant was treated with an equal volume phenol: chloroform: isoamyl alcohol (25 : 24 : 1) solution to extract proteins, and then the soluble phase was precipitated with 2.6 volumes 100% ethanol. After centrifugation at 14 000 r.p.m. for 30 min, the pellet was dissolved with nuclease-free water. *EGR1* transcripts were converted into cDNA and quantified using a OneStep RT-PCR kit (Qiagen) and a pair of primers (electronic supplementary material, table S1), visualized on native PAGE gels, and then were quantified using Image J. For the immobilized template assay, the same procedure was followed except for collecting the pellet and supernatant fractions at the desired time points. To map TOP2B, indicated restriction enzymes were added to the reactions at the appointed time points and allowed to sit for 15 min at 30°C. The pellet fraction was dissolved in 0.1 M Buffer D1. The pellet and supernatant fractions were visualized in PAGE gel (for DNA) and in SDS-PAGE, followed by silver staining (for proteins, silver nitrate purchased from Sigma) and western blotting (specific proteins of interest). Buffers used for transcription and immobilized template assays included freshly added complete protease inhibitors except for DTT.

### 2.8. Western blot and immunoprecipitation

Primary antibodies for probing phosphorylated S2 Pol II were obtained from Abcam (ab5095). The primary antibodies obtained from Bethyl Laboratories were as follows: BRCA1 (A300-000A), BARD1 (A300-263A), phosphorylated BRCA1 at S1524 (A300-001A), MED23 (A300-425A) and TOP2B (A300-949A). The antibodies for α-tubulin (sc-8035), TOP2B (sc-25330), TFIID (sc-421), TFIIF (sc-37430), TFIIE*α* (sc-133065), CDK9 (sc-13130) and ubiquitin (sc-8017) were from Santa Cruz Biotechnology. ELK1 (no. 91825) and Pol II (no. 2629) were purchased from Cell Signaling Technology. Each antibody was diluted in blocking solution within the range 1 : 500–1 : 3000, following the manufacturer's suggestion and empirical outcome. Rabbit and mouse secondary antibodies and luminol reagents for western blotting (sc-2048) were purchased from Santa Cruz Biotechnology and diluted to 1 : 2000 in blocking solution for usage. For western blotting, HEK293 lysates were collected using RIPA buffer (Cell Signaling Technology), and the protein concentrations of the lysates were quantified through Bradford assays (Bio-Rad) before SDS-PAGE to compare the samples in equal amounts of total proteins. For immunoprecipitation, protein A or G agarose beads (Cat. no. 20333, Pierce; sc-2002, Santa Cruz Biotechnology) were equilibrated with 0.15 M HEGN (50 mM HEPES, pH 7.6, 0.15 M KCl, 0.1 mM EDTA, 10% glycerol, 0.02% NP40), and TOP2B antibodies (A300-949A, Bethyl) or control IgG (sc-69876, Santa Cruz Biotechnology) were bound to the beads. The antibody-beads complex was washed with a 30-bead volume 0.5 M KCl-HEGN three times each and then with the same volume 0.15 M HEGN twice. The TOP2B antibody-bound protein A beads were incubated with 5 mg HeLa NE for 3 h at 4°C. The WT, SA, and SD BRCA1 proteins and control with a protein storage buffer only were added along with HeLa NE. The bead-protein complexes were washed with a 33-bead volume of 0.25 M KCl-HEGN three times. All buffer solutions included fresh complete protease inhibitors described above. The resultant pellet fractions were subjected to silver staining and western blotting analyses.

### 2.9. Chromatin immunoprecipitation and qPCR

The ChIP experiment was conducted following the Abcam X-ChIP protocol, with mild modifications. Cell lysis buffer included 5 mM PIPES (pH 8.0), 85 mM KCl, 0.5% NP-40. Nuclei lysis buffer, including 50 mM Tris-HCl (pH 8.0), 10 mM EDTA and 1% SDS, was added before sonication. Sonication was performed on the ice at 25% amplitude for 30 s at 2 min intervals (Vibra-Cell Processor VCX130, Sonics) and was optimized to produce DNA segments ranging between +100 and +1000 bp on a DNA gel. The cell and nuclei lysis buffers included the fresh protease inhibitors described above. The antibodies used in immunoprecipitation were Pol II (ab817, Abcam; no. 2629, Cell signaling; A304-405A, Bethyl Laboratories), phosphorylated S2 Pol II (ab5095, Abcam), BARD1 (A300-263A, Bethyl Laboratories), TOP2B (A300-949A, Bethyl Laboratories; sc-25330, Santa Cruz Biotechnology), BRCA1 (A300-000A, Bethyl Laboratories; sc-6954, Santa Cruz Biotechnology), phosphorylated BRCA1 (S1524) (A300-001A, Bethyl Laboratories; NB100-200, Novus Biologicals) and IgG (sc-2025, Santa Cruz Biotechnology). After IP and reverse cross-linking, the DNA was purified with a PCR purification kit (Qiagen). The ChIP products were analysed using real-time PCR using SYBR Green Realtime PCR Master Mix (Applied Biosystems) under thermal cycling as 1 min at 95°C followed by 45 cycles of 15 s at 95°C, 15 s at 55°C and 45–60 s at 72°C. The results were presented as per cent input or as relative enrichment to the input or IgG control. The primers used for the quantification are listed in electronic supplementary material, table S1.

### 2.10. Immunofluorescence

The HeLa cells were grown on a cover-glass and were cultured for 24 h in serum-containing medium. The cells were treated with etoposide 5 µM for 1 h. For immunofluorescence analyses, the cells were fixed with 4% para-formaldehyde for 20 min and washed twice with PBS. Then, the cells were permeabilized with 0.1% Triton X-100 in PBS. After blocking with 5% bovine serum albumin with PBS, the cells were incubated with anti-TOP2B (H-8, Santa Cruz Biotechnology) and anti-phospho-BRCA1 (Ser1524) (no. 9009, Cell Signaling Technology) antibodies for 3 h and secondary antibodies (anti-mouse or anti-rabbit) conjugated with Alexa Fluor 488/594 for 1 h. The nuclei were further stained with DAPI (blue, nuclear staining). Then, the fluorescence images were captured using an LSM 700 laser scanning confocal microscope with an objective C-Apochromat 40 × /1.2 W Corr UV-VIS-IR M27 (Zeiss). Fluorophores were visualized using the following filter sets: 488-nm excitation and band-pass 420–550 nm emission filter for Alexa 488; 555-nm excitation and long-pass 560 nm filter for Alexa 594. DAPI was visualized using 405 nm excitation and 410 nm emission long-pass filters. The scale bar on the bottom right of each image indicates 5 µm. The fluorescence intensity of pBRCA1 was measured using ImageJ (National Institutes of Health). The nuclei were selected using Freehead selection tool and then analysed.

### 2.11. *In vitro* ubiquitination assay

*In vitro* ubiquitination assay with recombinant TOP2B, WT/SA/SD BRCA1 and BARD1 proteins was conducted using recombinant human ubiquitin (U-100H, R&D system, USA) and HeLa NE as a source of E1 and E2 ligases in 20 mM Tris-HCl pH 7.5, 0.2 mM DTT, 5 mM MgCl_2_, 10 µM ZnCl_2_, 3 mM ATP, 1 mM BSA and the complete protease inhibitors. One reaction included 40 µM ubiquitin and 4 µg of HeLa NE when indicated. Ubiquitination was allowed for 3.5 h at 30°C. The reaction was stopped by the addition of 8X SDS loading buffer, and unmodified and ubiquitinated recombinant TOP2B was detected using His antibody (sc-8036, Santa Cruz biotechnology). For E2 ubiquitin-conjugating enzyme screening, E2 enzymes were purchased (ab139472, Abcam) and the experiments were performed according to the manufacturer's recommendations. Each of 11 E2 enzymes of UBCH1, UBCH2, UBCH3, UBCH5a, UBCH5b, UBCH5c, UBCH6, UBCH7, UBCH8, UBCH10 and UBCH13/MMS2 was added to 2.5 µM. The *in vitro* ubiquitination buffer A (10X) including 200 mM Tris-HCl, 2 mM DTT, 100 µM ZnCl_2_ and 10 mg ml^−1^ BSA was purified using 0.45 µm filter (Corning, USA) or the company provided buffer (*in vitro* ubiquitination buffer B, ab139472, Abcam, USA) was used to optimize the reactions and for the results shown in figure 5*d*. For UBCH2, the buffer provided with the enzyme (ab139472, Abcam) was used for its DTT sensitivity. E1 enzyme provided with the kit (ab139472, Abcam, USA) and ubiquitin (U-100H, R&D system) were added to 100 nM and 2 µM, respectively. Approximately 50 nM of the recombinant SA BRCA1-BARD1 complex as an E3 ligase was added to each reaction. Recombinant full-length hTOP2B as a target protein was added at 133 nM. The ubiquitination reaction was initiated by adding Mg^2+^/ATP (ab139472, Abcam), was allowed for 4 h at 37°C before the addition of 8X SDS gel loading buffer and was subjected to SDS-PAGE and immunoblotting.

### 2.12. Electrophoretic mobility shift assay

Full-length hTOP2B was diluted to targeted concentrations using TOP2B dilution buffer (10 mM HEPES, 130 mM KCl, 7.5 mM MgCl_2_, 5% glycerol, 10 µM ZnCl_2_ and 0.1% NP40). For each reaction, TOP2B was incubated with 100 ng DNA in TOP2B-DNA-binding buffer (13 mM HEPES, 98 mM KCl, 65 µM EDTA, 3.3% glycerol, 6.5 µM ZnCl_2_, 3.26 mM MgCl_2_ and 0.05% NP40) for 40 min at room temperature. A 5% native polyacrylamide gel was made using TBE buffer and pre-run in 0.5 X TB buffer for greater than 30 min. Before loading onto the gel, 50% glycerol was added to each sample to 13% as a final concentration. The gel was silver-stained (Cat. 209139, Sigma-Aldrich) and the band intensity was quantified using ImageJ. *K*_d_ values with 95% confidence intervals were calculated using Prism 8 (GraphPad, Inc.).

### 2.13. Statistical analysis

One- or two-way ANOVA was used to determine significance (*p* < 0.05) for ChIP-qPCR and qRT-PCR. *p*-values and graphs were calculated and drawn using Prism 8 (GraphPad, Inc.).

### 2.14. Bioinformatics

BRCA1 (GSM997540), TOP2B (GSM2442946) and input control (GSM2442939) ChIP-seq data were downloaded from the gene expression omnibus (GEO) database [45,46]. The raw data in the FASTQ format were processed using the Octopus-Toolkit (version 2.1.3) [47]. Briefly, the sequenced reads were trimmed using Trimmomatic (version 0.36) [48], and then were aligned to the reference genome (hg38 assembly) using HISAT2 (version 2.1.0) [49]. BRCA1 and TOP2B-binding sites (peaks) were identified using HOMER (version 4.10.1) [50] according to the following parameters: -region -size 500 -minDist 1500 -L 2 -LP 0.05 -localSize 15 000. Integrative Genomics Viewer (version 2.3.69) [51] was used to capture snapshots of the given loci with the BRCA1 or TOP2B ChIP-seq data. Heatmaps were generated for binding regions of the given factors using Deeptools (version 2.0) [52].

## 3. Results

### 3.1. BRCA1 is enriched in hIEGs and is phosphorylated at S1524 during transcriptional activation

We previously found that the DDR is coupled with productive transcriptional elongation in stress-inducible genes including *HSP70* and hIEGs and that the resulting DDR can be attributed, at least in part, to the catalytic activity of TOP2B [3,10]. Therefore, we hypothesized that TOP2B-mediated DSBs occur due to transcriptional activation and the DDR is a manifestation of the effort to repair the transcription-coupled DSBs. In spite of the involvement of the DNA-PK and Ku proteins, it is not yet clear which pathway among HDR, NHEJ, and non-canonical NHEJ is the primary repair mechanism for the TOP2B-mediated DSB. Notably, recent studies have also reported that DSB can be repaired by HDR in any cell cycle phase including, G_1_ [53–55], suggesting possible competition between the two repair pathways for the TOP2B-mediated DSB lesion in transcription.

**Figure 1.**
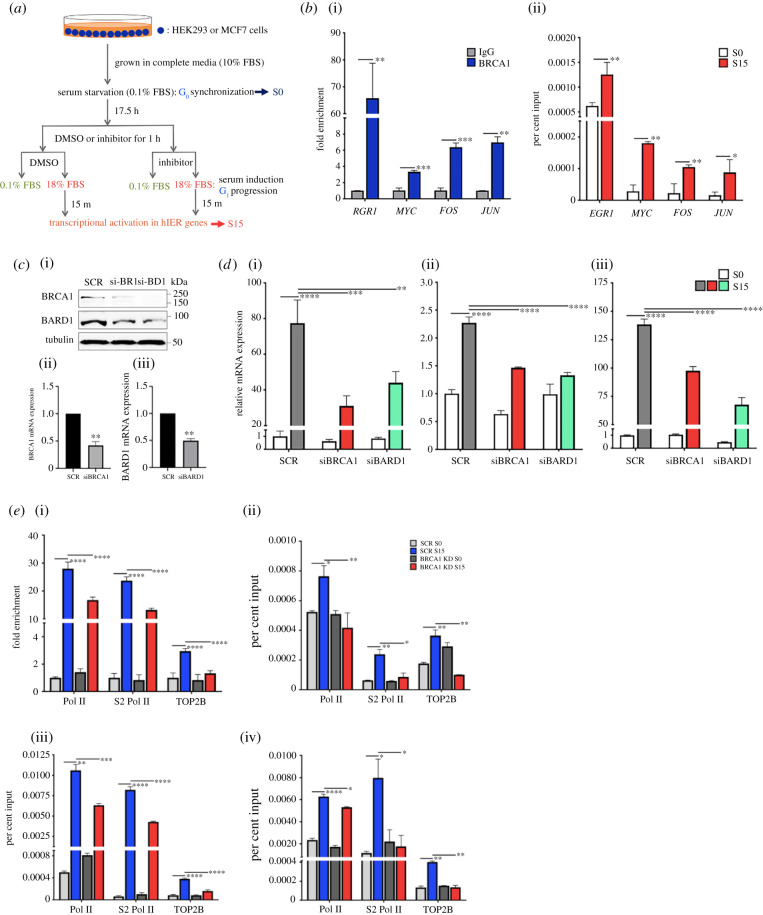
BRCA1 regulates serum-induced transcriptional activation. (*a*) Schematic overview of cell cycle synchronization and serum-induced transcriptional activation in hIEGs with or without chemical kinase inhibitors. FBS, fetal bovine serum. (*b*) ChIP-qPCR showing BRCA1 (i) and pBRCA1 (ii) occupancy at *FOS*, *MYC*, *JUN* and *EGR1*. S0, serum-starved cells at G_0_; S15, serum-induced cells. Error bars show standard deviations (s.d., *n* = 3). ****p < 0.0001, ***p < 0.001, **p < 0.01, *p < 0.05. (*c*) BRCA1 and BARD1 KD using siRNA species. (i), immunoblotting results showing the protein level of BRCA1, BARD1 and α-tubulin (tubulin, loading control, 70 µg cell lysate/lane) using siRNA species targeting BRCA1 (si-BR1) or BARD1 (si-BD1). SCR, scrambled siRNA. (ii, iii), BRCA1 and BARD1 mRNA expression in SCR control versus BRCA1 and BARD1 KD cells. Error bars show s.d. (*n* = 3). **p < 0.005. (*d*) qRT-PCR data showing the effects of BRCA1 or BARD1 KD on *EGR1* (i), *JUN* (ii) and *FOS* (iii) mRNA expression. β-actin was used as a reference gene. Error bars show s.d. (*n* = 3). ****p < 0.0001, ***p < 0.001, **p < 0.01. (*e*) ChIP-qPCR showing impaired Pol II, S2 Pol II and TOP2B recruitment upon gene activation at *EGR1* (i), *MYC* (ii), *FOS* (iii) and *JUN* (iv) in BRCA1 KD cells. Error bars show s.d. (*n* ≥ 3). ****p < 0.0001, ***p < 0.001, **p < 0.01, *p < 0.05.

To understand how TOP2B-mediated DSB formation and repair are regulated during transcriptional activation, we investigated whether BRCA1 is involved in the expression of stress-inducible genes. Using a previously established method [3,56], HEK293 cells were synchronized at the G_0_ phase of the cell cycle by serum starvation (S0) and then were allowed to progress into the G_1_ phase by serum supplement for 15 min (S15; figure 1*a*). The occupancy of BRCA1 at representative hIEGs, including *JUN*, *FOS*, *MYC* and *EGR1,* was examined using ChIP-qPCR. The data showed that BRCA1 was enriched in the TSSs of the tested genes (figure 1*b*). Then, we questioned whether BRCA1 is activated during transcriptional activation and measured the phosphorylation of BRCA1 at S1524 (pBRCA1), which is a signature DDR modification mediated by ATM and ataxia-telangiectasia and Rad3-related (ATR) proteins [23,42,57]. The level of pBRCA1 on the above hIEGs was probed before and after the serum-induced transcriptional activation through ChIP-qPCR analysis. The result showed an increased level of pBRCA1 on all tested genes, suggesting a possible DNA damage-induced activation of BRCA1 during processive transcription in hIEGs (figure 1*b*).

### 3.2. BRCA1 and BARD1 are important for the expression of hIEGs

Next, we examined whether BRCA1 is important for the expression of hIEGs. Therefore, BRCA1 was knocked down (KD) using siRNA species to reduce its expression at the mRNA and protein levels (figure 1*c*). In addition, we also knocked down BARD1 because this protein functions in a heterodimer with BRCA1 [25]. BARD1 KD decreased the protein levels of not only BARD1 but also BRCA1 (figure 1*c*), supporting a mutually interdependent requirement for stability between the two proteins [58]. Importantly, either BARD1 or BRCA1 KD decreased the mRNA levels of the representative hIEGs including *EGR1*, *JUN* and *FOS*, compared to the scrambled control when the gene expression was stimulated by serum (figure 1*d*). In addition, ChIP-qPCR showed that BRCA1 KD clearly decreased the level of total Pol II, serine 2-phosphorylated CTD of Pol II (S2 Pol II), and TOP2B on these genes, indicating reduced transcriptional activity in the gene despite serum induction (figure 1*e*). These data demonstrate that BRCA1 and BARD1 are important for the transcription of hIEGs.

### 3.3. BRCA1 phosphorylation at S1524 is important for active transcription at *EGR1*

**Figure 2.**
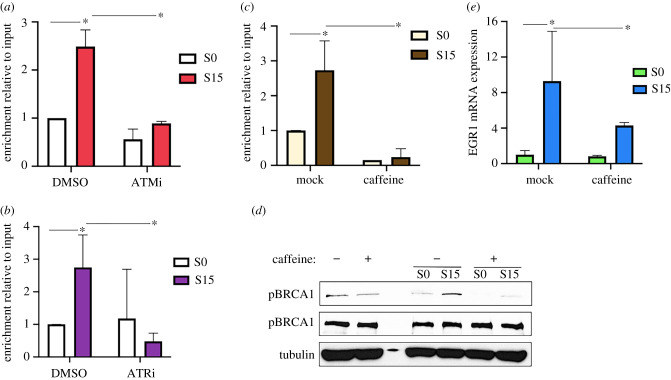
BRCA1 is phosphorylated by ATM and ATR upon transcriptional activation. (*a*) ChIP-qPCR of pBRCA1 with or without KU55933 (ATMi) in the *EGR1* TSS. Error bars show s.d. (*n* = 2). *p < 0.05. (*b*) ChIP-qPCR of pBRCA1 with or without VE-821 (ATRi) in *EGR1* TSS. Error bars show s.d. (*n* = 2). *p < 0.05. (*c*) ChIP-qPCR showing that caffeine, which inhibits both ATM and ATR, alleviates the accumulation of pBRCA1 on *EGR1* TSS upon transcriptional activation. Error bars show s.d. (*n* = 3). *p < 0.05. (*d*) Immunoblotting showing that caffeine reduces the cellular level of pBRCA1 overall and upon transcriptional activation. α-tubulin was used as a loading control. (*e*) qRT-PCR showing the reduction of *EGR1* mRNA level in caffeine-treated HEK293 cells upon transcriptional activation. β-actin was used as a reference and normalizer. Error bars show in s.d. (*n* = 3). *p < 0.05.

To understand the function of BRCA1 phosphorylation at residue S1524 in transcription, we used small molecule inhibitors of the ATM and ATR kinases, which are known to phosphorylate this residue during DDR signalling [23,42]. ATM inhibitor (KU55933) and ATR inhibitor (VE821) were added for 1.25 h before and during the serum induction (figure 1*a*). Our ChIP-qPCR data showed that exposure to either of these inhibitors reduced pBRCA1 levels in S15 samples in the TSS of *EGR1* (figure 2*a,b*). Consistent with these findings, caffeine, a compound that inhibits the kinase activities of both ATM and ATR [59], interfered with pBRCA1 accumulation at the gene upon transcriptional activation (figure 2*c*). In addition, the level of pBRCA1 was quantified by immunoblotting both with and without cell cycle synchronization. The experiment showed that caffeine treatment reduced levels of pBRCA1, confirming that these kinases are jointly responsible for BRCA1 phosphorylation (figure 2*d*). Consistently, caffeine decreased the *EGR1* mRNA level upon serum induction (figure 2*e*). These data suggested that BRCA1 is phosphorylated by ATM and ATR during transcriptional activation and that pBRCA1 is important for the gene expression of *EGR1*.

**Figure 3.**
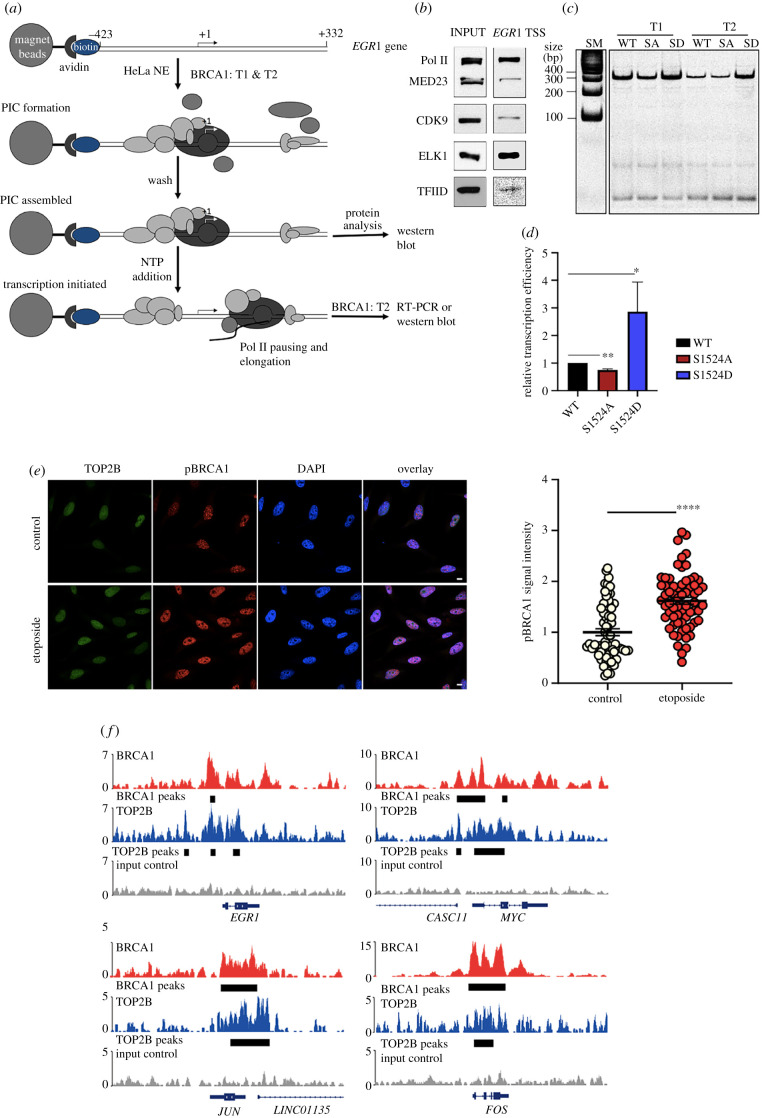
BRCA1 phosphorylation at S1524 is important for transcriptional activation. (*a*) Schematic overview of immobilized template and *in vitro* transcription assays. (*b*) Validation of PIC formation on the *EGR1* TSS (–423 to +332) using immunoblotting. (*c*) *In vitro* transcription assay using recombinant WT, S1524A (SA), and S1524D (SD) BRCA1 showing that SD activates *EGR1* transcription. (*d*) Quantification of the efficiency of S1524A and S1524D BRCA1 mutant proteins in stimulating transcription, relative to that of WT BRCA1. Error bars show s. d. (*n* = 3). **p < 0.002, *p < 0.02. (*e*) Immunofluorescence-confocal microscopy results showing increased levels of TOP2B and pBRCA1 in nuclei upon etoposide treatment. pBRCA1 intensity was quantified. *n* = 65. ****p < 0.0001. (*f*) Chromosome views of total BRCA1 (red) and TOP2B (etoposide-trapped, blue), and input control (grey) on representative hIEGs, *EGR1*, *JUN*, *MYC* and *FOS*, illuminating genomic colocalization and functional collaboration. Black bars indicate binding sites of a given factor, identified by HOMER with a false discovery rate (FDR)-adjusted *p*-value of 0.001.

We then sought to validate the functional importance of BRCA1 phosphorylation at S1524 for transcriptional activation using biochemical assays. For this purpose, recombinant WT BRCA1 (220 KDa) and two BRCA1 S1524 mutants, S1524A (phospho-null, SA) and S1524D (phosphomimeric, SD) were cloned and purified from *E. coli* and confirmed by immunoblotting (electronic supplementary material, figure S1A–C). The resultant WT, SA and SD BRCA1 proteins were compared using the *in vitro* transcription assay, which was established in our previous study [4]. In brief, transcriptional PIC was formed on a biotinylated *EGR1* template DNA construct that included the promoter and TSS (–423 to +332, electronic supplementary material, figure S2A) using HeLa NE (electronic supplementary material, figure S2B). For the *in vitro* biochemical analyses, including immobilized template and transcription assays, we used HeLa NE because it has been validated for these assays in the studies of ours and others (figure 3*a*) [4,60,61]. PIC formation on the *EGR1* TSS template was confirmed by the immobilized template assay and western blotting showing a promoter-binding transcriptional factor specific to the *EGR1* gene, ELK1 [62], and general transcription factors such as TFIID, CDK9 and Pol II (figure 3*a*,*b*). WT and mutant BRCA1 proteins were added along with NE only (T1) or both with NE and 3–5 min after NTP (T2; figure 3*a*). We hypothesized that the recombinant BRCA1 proteins supplemented at T1 could affect PIC formation and transcriptional initiation, while the proteins at T2 could influence both PIC formation and transcriptional initiation and promoter-proximal pausing and pause release. As an alternative to the use of radioactive rNTP and sequencing gel electrophoresis, *EGR1* transcripts from each experimental condition were converted into cDNA and quantified by PCR, using a pair of *EGR1*-specific oligonucleotides, amplifying from +1 to +332 (electronic supplementary material, table S1). Both T1 and T2 addition of the SD BRCA1 stimulated transcription relative to WT and SA, and yet the effect was more noticeable at T2 (figure 3*c*). In addition, to exclude any pre-existing *EGR1* transcripts in NE from being included in the quantification (electronic supplementary material, figure S2C), we modified 14 nucleotides from the template DNA between +140 and +160 to generate a mutant *EGR1* transcript (electronic supplementary material, figure S3A) that would be distinguished from the native one by the qPCR primers (electronic supplementary material, figure S3B). When the nascent transcript levels from the modified *EGR1* were compared among the recombinant BRCA1 species at T2, the results confirmed that phosphomimetic SD BRCA1 enhances transcription, but WT or SA do not (figure 3*d*; electronic supplementary material, figure S3C). SD showed the highest transcriptional activation, by approximately over 2.5-fold compared to the WT control (figure 3*d*). Together with the cell-based data shown in figures 1 and 2, these results strongly suggested that phosphorylation of BRCA1 at S1524 is important for the active transcription of the *EGR1* gene.

### 3.4. BRCA1 and etoposide-trapped TOP2B are colocalized in the TSSs of a large number of protein-coding genes

**Figure 4.**
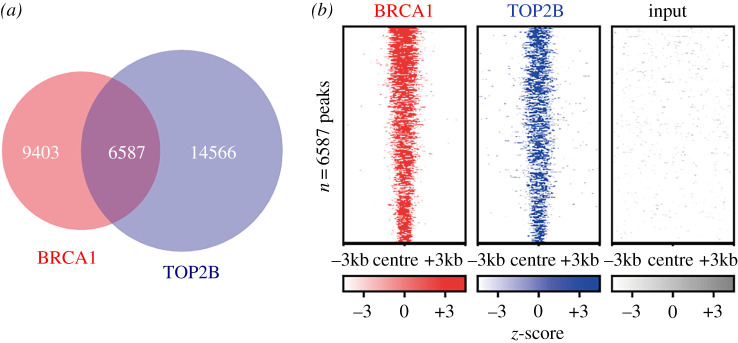
TOP2B active (etoposide-captured) and BRCA1-binding sites are largely overlapped genome-wide. (*a*) Venn diagram showing the overlap of BRCA1 and TOP2B-binding sites in MCF10A cells. (*b*) Heat maps of the genes co-occupied by TOP2B and BRCA1 (*n* = 6587).

Although BRCA1 is involved in the transcription of a diverse group of genes, the mechanisms through which it functions in transcription are incompletely understood [63,64]. Previous studies have shown that BRCA1 phosphorylation at S1524 is induced by DNA damage [23] and that the representative hIEGs listed above require DNA breaks mediated by TOP2B for Pol II pause release and active Pol II elongation [3,7]. Notably, a recent study found that BRCA1 regulates the resolution of TOP2B-DNA adducts during the transcriptional activation of oestrogen receptor-activated genes in the G_1_ phase [22]. Another study showed that the function of TOP2A, which is highly homologous to TOP2B except in its C-terminal domain [65], is regulated by the ubiquitin ligase function of the BRCA1-BARD1 complex during S phase [66]. Therefore, we attempted to examine the relation between TOP2B and BRCA1. We asked whether TOP2B-DNA adducts induced by etoposide increase pBRCA1 when HEK293 cells were treated with etoposide at 5 µM for 1 h. The data show that pBRCA1 is significantly increased in the nucleus of TOP2B-poisoned cells (figure 3*e*; electronic supplementary material, figure S4A), suggesting that DSBs caused by the abortive catalysis of TOP2B lead to phosphorylation of BRCA1. In addition, we examined specific locations of BRCA1 and TOP2B in hIEGs using the ChIP-seq data (GSM2442946 and GSM997540) [45,46]. The colocalization of BRCA1 and TOP2B was evident on the representative hIEGs, including *EGR1*, *JUN*, *MYC* and *FOS* (figure 3*f*). Furthermore, the occupancy of both BRCA1 and TOP2B trapped by etoposide showed a notable colocalization in a large number of protein-coding genes (*n* = 6587; figure 4; electronic supplementary material, data S1). These results therefore suggested a potential functional relationship between TOP2B and BRCA1 whereby these proteins might cooperate to regulate the transcription of a subset of genes.

### 3.5. BRCA1-BARD1 complex interacts with and stabilizes TOP2B

**Figure 5.**
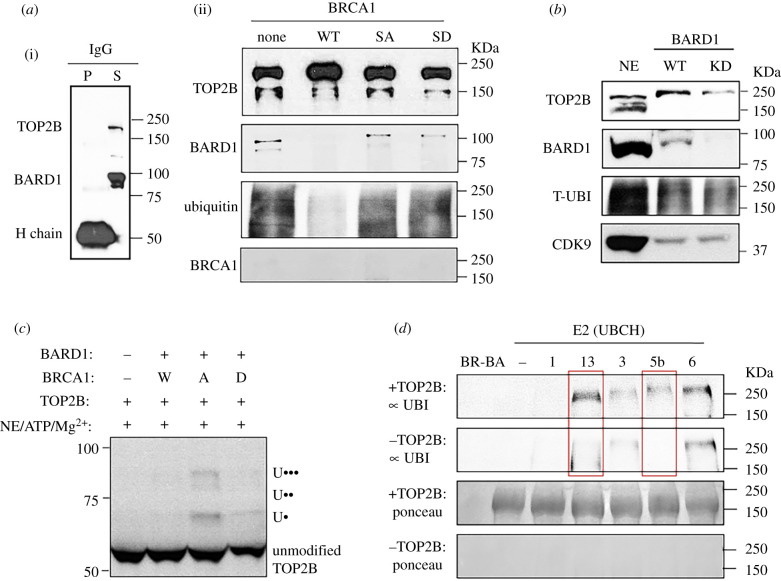
BRCA1-BARD1 ubiquitinates TOP2B in a phosphorylation-dependent manner. (*a*) (i) Immunoprecipitation with control IgG against 5 mg of HeLa NE. P, pellet (bound) and S, supernatant (unbound) fraction. Pellet and supernatant were loaded at 1 : 10 and 1 : 100 inputs, respectively. H chain, IgG heavy chain. (ii) Immunoprecipitation of TOP2B antibody against HeLa NE, followed by immunoblotting. (*b*) Immunoblotting data showing that BARD1 KD decreases the level of TOP2B proteins and ubiquitination. CDK9 was used as a loading control. HeLa NE was included as a technical control. (*c*) *In vitro* ubiquitination assay followed by immunoblotting showing discrete bands at about 56, 68, 81 and 91 KDa for 0, 1 (U•), 2 (U••) and 3 (•••) ubiquitin proteins ligated to TOP2B^1–566^ by SA BRCA1-BARD1. W, WT BRCA1; A, SA BRCA1; D, SD BRCA1. (*d*) *In vitro* ubiquitination assay and immunoblotting screening the E2 enzymes, UBCH1, UBCH3, UBCH5b, UBCH6 and UBCH13/MMS2 for the ubiquitination of TOP2B^deubi^ in collaboration with SA BRCA1-BARD1. Red boxes indicate UBCH5b and UBCH13/MMS2 to collaborate with the BRCA1-BARD1 complex to ubiquitinate TOP2B.

We hypothesized that the BRCA1-BARD1 complex might be involved in TOP2B regulation in hIEGs. To test this hypothesis, we sought to determine whether TOP2B and the BRCA1-BARD1 complex physically interact using immunoprecipitation and immunoblotting. The control experiment with IgG did not pull down the proteins of interest from HeLa NE (figure 5*a*). The results using TOP2B-specific antibodies showed a strong and stable interaction between TOP2B and BARD1 that was sustained through a series of stringent high-salt washes with 0.25 M HEGN. However, little recognizable binding between BRCA1 and TOP2B proteins was detected under these conditions (figure 5*a*). Because BRCA1 forms a heterodimer with BARD1 in the cell at a physiological salt concentration of about 0.15 M, it is suggested that TOP2B interacts with the BRCA1-BARD1 complex through its stable association with BARD1. Intriguingly, recombinant WT BRCA1, added to the IP along with NE, sequestered BARD1 from TOP2B, apparently interfering with their interaction. By contrast, the SA and SD BRCA1 proteins did not interfere with the interaction between BARD1 and TOP2B, which was sustained almost to a level comparable to that of the control (figure 5*a*). It was noticed that BARD association with TOP2B yielded ubiquitination signals that probably pertained to TOP2B. When recombinant WT BRCA1 was supplemented in the reaction, BARD1 dissociated from TOP2B, resulting in little ubiquitination signal. By contrast, neither the SA nor the SD BRCA1 proteins interfered with the BARD1-TOP2B interaction, and ubiquitination signals were observed in both samples although they exhibited different band patterns (figure 5*a*). These results suggested the possibility that BRCA1 could regulate the physical interaction of BARD1 with TOP2B and that the S1524 site of BRCA1 might be important in regulating the BARD1-TOP2B interaction required for TOP2B ubiquitination.

To further investigate the functional relationship between TOP2B and BARD1, BARD1 was knocked down and the levels of TOP2B protein were examined, comparing the NEs from WT and BARD1 KD cells. The data showed a notable reduction of TOP2B in BARD1 KD cells, which suggested TOP2B stability is dependent on BARD1 (figure 5*b*; electronic supplementary material, figure S5A). In addition, the total ubiquitination signals of proteins with electrophoretic mobilities between 150 and 250 KDa were mildly reduced in the BARD1 KD cells (figure 5*b*). Although the source of the ubiquitin signals cannot be distinguished from this experiment, and it is unclear which proteins are modified, ubiquitination became reduced in a manner that correlated with the protein levels of BARD1 and TOP2B (figure 5*a*,*b*; electronic supplementary material, figure S5A). We interpreted these findings to indicate that either: (i) cellular TOP2B is present in an ubiquitinated form, so that TOP2B protein reduction itself results in the decreased ubiquitin signals; or (ii) ubiquitination may regulate TOP2B stability so that the reduced ubiquitination leads to a decline in protein level in BARD1 KD.

### 3.6. BRCA1-BARD1 ubiquitinates TOP2B in collaboration with UBCH5b and UBCH13/MMS2

Therefore, we asked whether the BRCA1-BARD1 complex could ubiquitinate TOP2B as an E3 ligase and tried to identify the role of BRCA1 phosphorylation at S1524 in TOP2B ubiquitination through *in vitro* ubiquitination analyses. Recombinant TOP2B containing 1–566 amino acids (TOP2B^1-566^) and full-length BARD1 were cloned and purified from bacteria and confirmed by immunoblotting (electronic supplementary material, figure S5B,C and table S1). To supply E1 and E2 enzymes, a small amount of NE was used. His^6^-tagged TOP2B^1–566^ was incubated with WT, SA or SD BRCA1 and BARD1 in the *in vitro* ubiquitination and the results were analysed by immunoblotting using an anti-His antibody as all commercial antibodies that specifically recognize TOP2B are produced using its C-terminal fragments as antigens. The controls, BRCA1-BARD1 complex or BARD1 only without TOP2B^1–566^ did not display the shifted band characteristic of ubiquitinated TOP2B^1–566^ (electronic supplementary material, figure S5D). Importantly, SA BRCA1 and BARD1 resulted in the formation of mono-, di- and tri-ubiquitinated TOP2B^1–566^, calculated from the log_10_MW plot of molecular migration (figure 5*c*). WT and SD BRCA1 were less capable of ubiquitinating TOP2B^1–566^ (figure 5*c*; electronic supplementary material, figure S5D). These results showed that the BRCA1-BARD1 complex ubiquitinates TOP2B, and this modification is dependent on the phosphorylation status of BRCA1 at S1524, as unphosphorylatable BRCA1 S1524 supports discrete ubiquitination of TOP2B^1–566^ (figure 5*a*,*c*).

To identify the E2 enzyme(s) that cooperates with BRCA1-BARD1 to ubiquitinate TOP2B, 11 E2 ubiquitin-conjugating enzymes (UBCH1, UBCH2, UBCH3, UBCH5a, UBCH5b, UBCH5c, UBCH6, UBCH7, UBCH8, UBCH10 and UBCH13/MMS2) were screened for their ability to ubiquitinate TOP2B *in vitro*. A full-length recombinant TOP2B expressed and purified from HEK293F cells displayed intact catalytic activity in the *in vitro* decatenation assay and was used for the ubiquitination assay (electronic supplementary material, figure S5E,F). Because the recombinant human full-length TOP2B obtained from HEK293F cells was partially ubiquitinated (hereafter, TOP2B^ubi^), we added the deubiquitinase USP2 in wash steps during the YFP-tag purification. Subsequent washes and FPLC purification removed USP2 to yield deubiquitinated full-length TOP2B (hereafter, TOP2B^deubi^; electronic supplementary material, figure S6A). We tested the purified E1 and E2 enzymes using the anti-His antibody and confirmed that there were no detectable signals over 150 KDa in which TOP2B protein is located (electronic supplementary material, figure S6B). TOP2B^deubi^ and TOP2B^ubi^ were used as substrates, non-phosphorylatable SA BRCA1-BARD1 was used as an E3 enzyme, and recombinant UBA1 was used as an E1 enzyme in *in vitro* ubiquitination assays. Our thorough screenings indicated that UBCH13/MMS2 and UBCH5b E2 enzymes yielded ubiquitinated proteins with masses that were related to TOP2B (figure 5*d*; electronic supplementary material, figure S6C, D). These distinguishable bands could be attributable to short or long ubiquitin chains attached to TOP2B. To exclude the possibility that these bands correspond to ubiquitinated E1, E2 or E3, we probed His^6^-tagged E1, E2 and E3 enzymes using the anti-His antibody to validate that the ubiquitination signals above 150 KDa could only be due to ubiquitinated TOP2B (electronic supplementary material, figure S6B). The ubiquitination bands were not observed when TOP2B or E1 enzyme were omitted, confirming that the ubiquitination is attributed to TOP2B (figure 5*d*; electronic supplementary material, figure S6C). These results suggest that the BRCA1-BARD1 complex can interact with the E2 enzymes, UBCH13/ MMS2 and UBCH5b to ubiquitinate TOP2B in different patterns, perhaps for distinctive functions or outcomes.

### 3.7. BARD1 and TOP2B colocalize in the *EGR1* TSS *in vitro*

**Figure 6.**
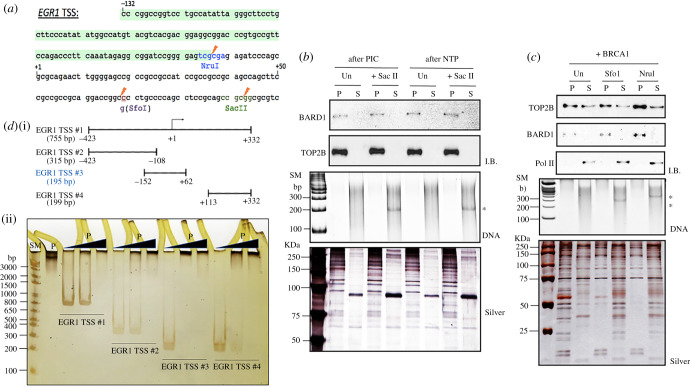
TOP2B and BARD1 bind to *EGR1* TSS between –132 and –15. (*a*) DNA sequence of *EGR1* TSS (–132 to +332). Orange flash signs indicate the restriction enzyme sites for NruI, SfoI and SacII used to map the factor binding region. Coloured letters indicate those that were subjected to mutations for the purposes indicated in the text. Altered sequences are presented under the original ones. TOP2B and BARD1 mutual binding site mapped in this study was boxed with light green. (*b*) Immobilized template assay combined with restriction enzyme digestion using *EGR1* TSS (–423 to +332) and SacII (Sac, to digest at +92), followed by immunoblotting. SacII added immediately after PIC formation or NTP addition. Un, undigested template DNA; I.B., immunoblotting probing BARD1 and TOP2B; DNA, native PAGE detecting the released DNA fragment (241 nt) after SacII digestion; Silver, silver staining visualizing the proteins bound on the DNA. P, pellet; S, supernatant fraction. (*c*) Immobilized template assay using the *EGR1* template (−132 to +332) in the presence or absence of BRCA1, combined with restriction enzyme digestion with NruI and SfoI that cut at −15 and +68, respectively. Recombinant WT BRCA1 was added at T2, immediately before the template was digested by restriction enzymes. The pellet and supernatant fractions were analysed by immunoblotting. (*d*) (i) Diagram of the four *EGR1* TSS fragments. (ii) EMSA followed by silver staining showing TOP2B^ubi^ binding to *EGR1* TSS fragments with different affinities. The strongest binding was observed with *EGR1* TSS no. 3 (–132 to +62). SM, size marker; P, TOP2B^ubi^. The second lane shows TOP2B^ubi^ only.

We next studied the dynamics of TOP2B and BRCA1-BARD1 complex interaction on the *EGR1* TSS using the immobilized template assay *in vitro*. Initially, the binding sites of these proteins on the TSS of the *EGR1* gene were investigated. The biotinylated *EGR1* template (–423 to +332, 120 ng per reaction) was first incubated with HeLa NE to assemble the PIC, and unbound or loosely associated proteins were washed off (figure 3*a*). Then, the complex was treated with or without the restriction enzyme SacII, which cuts the template at +92 (figure 6*a*,*b*). The digested DNA and proteins derived from these reactions were visualized using native PAGE and SDS-PAGE followed by silver staining, respectively, confirming the proper fragmentation of the template and proteins associated with each fraction (figure 6*a*,*b*). TOP2B was found solely in the pellet fraction, along with BARD1, as observed in immunoblotting (figure 6*b*). These proteins of interest did not bind to the beads-only control without the *EGR1* TSS template (electronic supplementary material, figure S7A). These data indicated that TOP2B and BARD1 bind to the TSS of *EGR1* between –423 and +92 (figure 6*a*,*b*). NTP addition after the PIC formation on the template did not lead to significant differences in these properties (figure 6*b*).

In addition, the association between TOP2B and the *EGR1* TSS was monitored in the presence of BRCA1. Recombinant WT BRCA1 was supplemented at T2. The addition of WT BRCA1 increased the total amount of TOP2B associated with the *EGR1* template (–132 to +332), compared to the samples without it (figure 6*c*; electronic supplementary material, figure S7B for without BRCA1 data). However, a noticeable fraction of TOP2B was released from the template and detected in the supernatant fraction. Interestingly, BRCA1 supplementation at T2 increased levels of Pol II in the supernatant fraction (figure 6*c*; electronic supplementary material, figure S7B). A SfoI site was introduced into the 25 bp upstream of +92 by a site-directed point mutation to further map the TOP2B-binding site (figure 6*a*; electronic supplementary material, figure S7C). The proteins in the pellet and supernatant fractions and the digested DNA released from the pellet fraction are shown in silver staining and native PAGE in figure 6*c*. SfoI and NruI digestion, which cut the template DNA at +68 and –15, respectively, indicated that TOP2B and BARD1 are stably associated with the regions between –132 and –15 (figure 6*c*; electronic supplementary material, figure S7B). Consistent with the IP results (figure 5), these experiments suggest the colocalization and physical interaction between TOP2B and BARD1 in the *EGR1* TSS. To assess the affinity between *EGR1* TSS and TOP2B, we performed a electrophoretic mobility shift assay (EMSA) using TOP2B^ubi^ and different fragments of *EGR1* template DNA (figure 6*d*; electronic supplementary material, figure S8A) Although a large amount of TOP2B bound to all the fragments tested, except for the single-stranded *EGR1* TSS, the EGR1 TSS no. 3 (–132 to +62) bound to TOP2B^ubi^ with the strongest affinity (figure 6*d*; electronic supplementary material, figure S8B).

### 3.8. Ubiquitination is required for effective binding of TOP2B to the *EGR1* TSS

**Figure 7.**
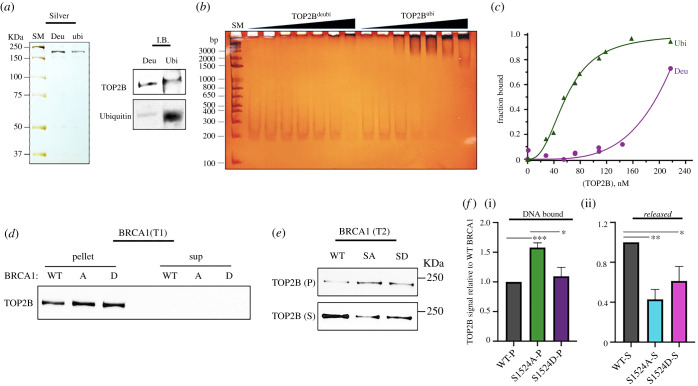
BRCA1 phosphorylation controls TOP2B ubiquitination and DNA-binding affinity. (*a*) Purified TOP2B^ubi^ (ubi) and TOP2B^deubi^ (Deu) shown by silver staining and immunoblotting. (*b*) EMSA comparing TOP2B^ubi^ versus TOP2B^deubi^ for their binding affinity to EGR1 TSS no. 3 (–132 to +62). Silver-stained. SM, DNA size marker. (*c*) Ubiquitinated TOP2B binds to DNA with much higher affinity than deubiquitinated one. A plot summarizing EMSA to derive *K*_D_ values. (*d*) Immobilized template assay results. Tight TOP2B association with *EGR1* TSS (Pellet, –423 to +332) before NTP addition, regardless of recombinant BRCA1 species added at T1 during PIC formation. (*e*) Immobilized template assay results. Comparison of the degrees of TOP2B associated (Pellet, P) with and dissociated from *EGR1* TSS (Supernatant, S) when WT, SA and SD BRCA1 were added after NTP addition (T2). (*f*) Quantification of TOP2B in pellet (DNA bound, i) and in supernatant (released, ii) in immobilized template assays. Error bars in s.d. (*n* = 3). ***p < 0.005, **p < 0.01, *p < 0.05.

We hypothesized that TOP2B ubiquitination might change its ability to bind to DNA. Therefore, TOP2B^deubi^ and TOP2B^ubi^ (Deu and Ubi in figure 7*a*, respectively) were incubated with 100 ng of *EGR1* TSS no. 3 in increasing concentrations (up to 217 nM) and the reactions were visualized using silver staining (figure 7*b*,*c*; electronic supplementary material, figure S5E, S6A, S8C,D). Strikingly, TOP2B^deubi^ was deficient for binding the DNA at concentrations below 150 nM (figure 7*b*,*c*; electronic supplementary material, figure S8D). The *K*_d_ value for TOP2B^ubi^ and EGR1 TSS no. 3 binding is 59.9 ± 6.1 nM (figure 7*c*; electronic supplementary material, figure S8C). These data suggested that TOP2B ubiquitination enhances its binding to *EGR1* TSS. To further understand the role of BRCA1 phosphorylation at S1524 in TOP2B association with the *EGR1* gene, we compared WT, SA and SD BRCA1 for TOP2B binding to the *EGR1* TSS in the immobilized template assay. Recombinant BRCA1 species were added at T1 or T2, and the pellet and supernatant fractions were separated without or with NTP addition, respectively. Regardless of WT and mutant BRCA1 proteins added during the PIC formation (T1), TOP2B was tightly bound to the *EGR1* template without NTP addition, which represents the stage before transcriptional initiation (figure 7*d*). Importantly, when BRCA1 species were added after NTP addition (T2), SA increased the level of TOP2B stably associated with the *EGR1* template to levels that exceeded WT BRCA1 and SD BRCA1 (figure 7*e*,*f*). Consequently, less TOP2B was released into the supernatant for the SA BRCA1 sample (figure 7*e*,*f*). Together with the biochemical data shown in figure 5*a*,*c*, and *d*, these results suggest that the residue S1524, which is located in proximity to the BRCA1 C-terminal domain specialized in the binding of phosphoproteins [67] could be critical for the BRCA1-BARD1 functional interaction. We interpret this finding to mean that un-phosphorylated BRCA1 (SA mutant) allows BRCA1-BARD1 to more effectively ubiquitinate TOP2B (figure 5*a–d*; electronic supplementary material, figure S5D), which stabilizes TOP2B association with the DNA (figure 7*a*–*f*; electronic supplementary material, figure S8B, C). WT and the phosphomimetic SD BRCA1, on the other hand, are less effective at stabilizing TOP2B on the DNA (figure 5*a*,*c*,*d*; 7*d*–*f*).

**Figure 8.**
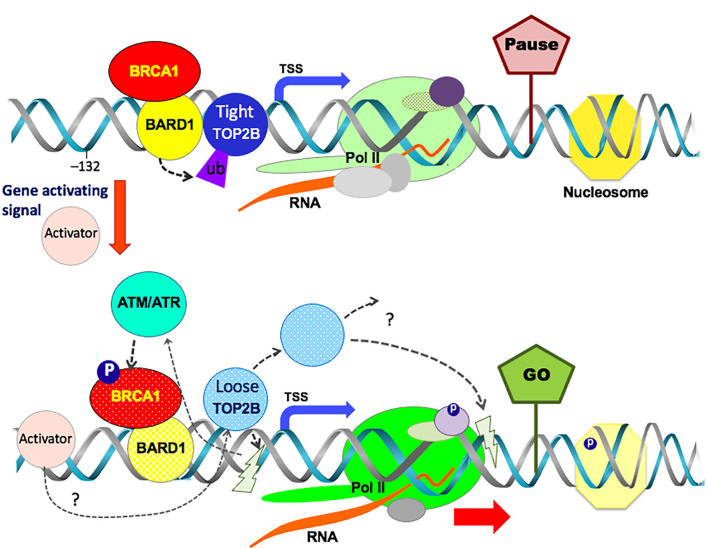
Model of the BRCA1-BARD1 complex-mediated TOP2B regulation. During the resting state of transcription, Pol II is paused in the promoter-proximal site in hIEGs. The pausing is induced and stabilized by various factors including transcription factors, nucleosome modifiers and nucleic acids. The BRCA1-BARD1 complex that is engaged with hIEGs interacts with and ubiquitinates TOP2B in the *EGR1* TSS. This ubiquitination (marked as ub) confers an enhanced DNA-binding affinity and stability to TOP2B (Tight TOP2B). In transcriptional activation, ATM/ATR phosphorylates its substrates including BRCA1 at S1524 (phosphorylation, marked as P in a blue circle). Phosphorylated BRCA1 alters the functional interaction between BRCA1-BARD1 and TOP2B to mitigate TOP2B ubiquitination. This event destabilizes and loosens TOP2B (Loose TOP2B) from the TSS. The interplay between BRCA1-BARD1 complex and TOP2B appears to be crucial in transcriptional regulation of hIEGs. Lightning marks are proposed catalysis sites of TOP2B.

Together, our cell-based, genomic, and biochemical analyses indicate a novel mechanism in which the BRCA1-BARD1 complex is involved in the transcription of hIEGs by modulating TOP2B. We suggest that in the resting state of transcription, BRCA1-BARD1 complex ubiquitinates TOP2B to promote stable binding to the TSS (figure 5*a*–*d*, 6*d*, 7*a*–*f*; electronic supplementary material, figure S5D, S6C,D). In transcriptional activation and pause release, ATM and ATR are activated to phosphorylate BRCA1 at S1524 (figures 1*b*, 2*a*–*d*). BRCA1 phosphorylation at S1524 controls the interaction between TOP2B and the BRCA1-BARD1 complex (figures 5*a*,*c*, 7*e*,*f*), resulting in decreased or differential TOP2B ubiquitination, which then destabilizes the TOP2B-DNA interaction (figures 7*a*–*f*, 8; electronic supplementary material, figure S8B–D).

## 4. Discussion

Our findings suggest that the BRCA1-BARD1 complex is important for serum-inducible transcription. We showed that BRCA1 binds near the TSS and is phosphorylated at S1524 during transcriptional activation of the *JUN*, *FOS*, *MYC* and *EGR1* genes (figures 1 and 2). BRCA1 binding to these genes could also be mediated through BARD1 for their heterodimeric interaction because BARD1 was associated with the DNA and TOP2B more stably in the *EGR1* TSS (figure 5*a*, 6*b*,*c*; electronic supplementary material, figure S7A,B). The phosphorylation of BRCA1 at S1524, mediated by ATM and ATR, is important for the active gene expression of *EGR1 in vivo* and *in vitro* (figures 1–3). Mechanistically, we suggest that BRCA1-BARD1 physically interacts with and ubiquitinates TOP2B (figure 5*a*,*c*,*d*; electronic supplementary material, figure S6C,D). The ubiquitination of TOP2B is important to modulate its stability and DNA association (figures 5*b*, 6*d*, 7*a*–*f*; electronic supplementary material, figure S8B–D) and the BRCA1-BARD1 complex functions as an E3 ligase for TOP2B (figure 5*c*–*e*; electronic supplementary material, figure S6C,D). The functional interaction between the BRCA1-BARD1 complex and TOP2B appears to be regulated by BRCA1 phosphorylation at S1524 (figures 5*a*,*c*, 7*d*–*f*; electronic supplementary material, figure S5D). Furthermore, the phosphorylation of BRCA1 S1524 controls the association between TOP2B and DNA (figure 7*e*,*f*). Unphosphorylatable BRCA1 mutated at S1524 augments TOP2B ubiquitination and strengthens TOP2B binding to the *EGR1* TSS (figure 5*a*,*c*,*d*, 7*b*–*f*; electronic supplementary material, figure S5D, S6C,D), suggesting a tighter DNA binding by ubiquitinated TOP2B in the resting state of transcription or RNA Pol II pausing in which BRCA1 S1524 is less phosphorylated. Supporting this inference, it was reported that TOP2B peaks are located coincidently with active and repressive histone markers and Pol II in MCF7 cells and then the treatment with estradiol reduces the peaks associated with active histone markers and Pol II [68]. We note that the type of ubiquitination may determine whether TOP2B is degraded [69], stabilized or modulated for its catalysis, a mechanism which awaits further studies.

In addition, we identified the E2 enzymes, UBCH13/MMS2 and UBCH5b, collaborating with the BRCA1-BARD1 complex to ubiquitinate TOP2B through the *in vitro* ubiquitination assays (figure 5*d*; electronic supplementary material, figure S6A–D). UBCH13/MMS2 is known to ubiquitinate proteins involved in signal transduction and for activating NF-κB [70]. On the other hand, UBCH5 enzymes, which are known to poly-ubiquitinate proteins including p53 and BRCA1 [71,72], appear to also poly-ubiquitinate TOP2B (figure 5*d*; electronic supplementary material, figure S6C,D). Further study is required to determine the ubiquitinated residues in TOP2B and the roles of these E2 enzymes in BRCA1-BARD1-mediated TOP2B ubiquitination.

We showed that BRCA1 colocalizes with TOP2B in a large number of genes including serum-inducible genes (figures 3*f*, 4; electronic supplementary material, figure S4B). Furthermore, we identified a high-affinity TOP2B-binding site (–132 to +62) in the *EGR1* TSS and showed that BARD1 is colocalized with TOP2B within the region (–132 to –15, figures 6*a*–*d*, 7*a*–*c*; electronic supplementary material, figure S7A,B). It is still unclear whether the TOP2B-DNA resolution site(s) is limited to the TSS or extended to the gene body, along with transcriptional activation and elongation. In fact, TOP2B and TOP2B-mediated DDR signalling during hIEG transcription occur in the entire promoter, TSS and gene body of transcriptionally activated genes [3]. In addition, a recent genomic study found that TOP2B-mediated DSB is mapped both in the promoter along with CTCF and in the promoter-proximal site and gene body [73] whereas another study without etoposide poisoning mapped TOP2B-binding sites to the promoter [7,74,75]. Interestingly, the TOP2B-mediated DSB sites in the TSS and gene body appear to be closely correlated with transcriptional activity and gene expression [3,73]. From these earlier and more recent data, it can be speculated that TOP2B is mainly associated with the promoter of a gene during the resting stage of gene expression, while its function might become extended to the TSS and gene body along with the promoter region during transcriptional activation. Validation of this hypothesis would be important, awaiting further study.

Because the DNA torsional stress during transcription requires TOP2B-mediated DSB for resolution, the fidelity of the enzyme for removing DNA supercoiling and immediately resealing the broken DNA ends is likely to be crucial. A recent study suggested that TOP2A, but not TOP2B, is responsible for regulating supercoiling without provoking DSB in the promoter of hIEGs and the inhibition of TOP2A activates transcription [76]. Although it is likely that TOP2A is poorly expressed in post-mitotic neuronal cells used for the study [77,78], this may suggest a potential competition or redundant function of TOP2A and TOP2B in the promoter while TOP2B functions at both TSS and gene body in hIEGs. Other studies have indicated the frequent and spontaneous formation of TOP2B-DNA adducts through abortive catalysis during the transcription of stimulus-inducible genes under normal physiological conditions [21,22], proposing that the prompt release and precise repair of TOP2B-DNA adducts is necessary to preserve genomic stability. Our current work suggests that the E3 ligase function of BRCA1-BARD1 controls TOP2B stability and binding to DNA in a BRCA1 phosphorylation-dependent manner. These findings raise important questions as to whether the BRCA1-BARD1 complex could be involved in the DNA repair of TOP2B-mediated DSB upon transcriptional activation, which are to be addressed in future.

We propose that TOP2B and BRCA1-BARD1 interaction regulates TOP2B ubiquitination and association with the TSSs of stress-inducible genes before gene activation (figure 8). In this model, transcriptional activation and Pol II pause release trigger the phosphorylation of BRCA1 by the PI3 kinase family proteins, ATM and ATR. Phosphorylated BRCA1 modulates the functional interaction between BARD1 and TOP2B, which reduces or alters TOP2B ubiquitination to loosen TOP2B from the TSS of the activated gene. Our findings present a novel physiological function for BRCA1-BARD1 complex and the mechanism by which the complex modulates TOP2B in transcription.

## Data accessibility

All data are available in the manuscript or the electronic supplementary material [79].
